# *AKT1*, *PRDM4*, and *BAX* are the natural markers of psychological endurance threshold

**DOI:** 10.1002/brb3.1306

**Published:** 2019-05-16

**Authors:** Zhijian Xu, Qicheng Jing, Houcan Zhang, Yue Liu

**Affiliations:** ^1^ Guangdong Provincial Government Office Guangzhou China; ^2^ Institute of Psychology Chinese Academy of Sciences Peking China; ^3^ Department of Psychology Beijing Normal University Peking China; ^4^ Department of Biochemistry and Molecular Biology, School of Basic Medical Sciences Southern Medical University Guangzhou China

**Keywords:** abnormal behavior, *AKT1*, *BAX*, psychological endurance threshold, *PRDM4*, social harm

## Abstract

**Introduction:**

Abnormal behavior can cause harm or loss to oneself, the family, and society and may be related to psychological endurance levels. With early identification and early intervention, the occurrence of harm can be prevented and the loss can be reduced. Now there is no clear definition of psychological endurance levels and no accurate measurement tools yet.

**Methods:**

This study first proposes the concept of psychological endurance threshold (PET) and defines that as: “the psychological state threshold of human objective physiological characteristics and outbreaks of abnormal behavior led by subjective cognitive level difference”. The study hypothesizes that human behavior is related to it, and constructs multiple measurement method tools to measure it.

**Results:**

Here we show PET exists objectively and can be measured exactly by methods such as psychological endurance threshold measurement table, experience evaluation, dopamine level detection, and genetic testing. In particular, PET is determined by AKT1, PRDM4, and BAX which are the natural markers of PET.

**Conclusions:**

The significance of this study is to discover people with abnormal expression of AKT1, PRDM4, and BAX who have lower PET and tend to commit abnormal behavior more easily. Understanding PET will enable people to make self‐adjustment or to intervene by professionals as soon as possible and in a timely manner in the face of various negative stimuli in work and life, especially for people with lower PET, people should intervene as early as possible to reduce the harm to the individual, family and society.

## 1 INTRODUCTION

We all know that people have a certain psychological endurance level, but what is its definition, what is the threshold, and how to measure it, have not been studied yet, there are only researches on the relationship between abnormal behavior and negative stimulation. People often attribute abnormal behavior to constant negative stimulation. If a certain level is reached or exceeded, abnormal behaviors will occur. Some abnormal behaviors can cause great harm or loss to the individual, family, and society. Negative things are also called negative stimuli. Previous studies have conducted relevant experiments to examine the properties and performance of negative stimuli.

Negative stimuli can lead to aversive emotional changes. Disgust is a negative emotional experience caused by unpleasant and annoying stimuli (Rozin, Haidt, & McCauley, 2000). Hoefling et al. (2009), Anderson and Platten (2011, p.463–466), Markovitch, Netzer, and Tamir (2016), andOlatunji, Ciesielski, Wolitzky‐Taylor, Wentworth, and Viar (2012) conducted experiments on people's feelings of disgust produced by feelings of deprivation and negative stimuli. The results showed that if proper intervention cannot be provided for negative events or negative stimuli, abnormal behavior will occur when these stimuli reach or exceed a certain level. Some abnormal behaviors can cause considerable harm.

Hegedüs et al. (2014), Schmitz, Vierhaus, and Lohaus (2013), Ryan, Kujawa, Hammill, Le Prell, and Kil (2016), and Szelényi A (Szelényi, Journée, Herrlich, Galistu, van den Berg, van Dijk, 2013) conducted human tolerance experiments on pain, noise, and electrical currents. Black, Gabbett, Cole, and Naughton (2016), Estes (2015, p. 1,174–1,187), and Neuraz et al. (2015) conducted experiments on people's workload tolerance. The results indicated that a tolerance level exists and it varies from person to person. Some researchers have concluded (Leyro, Zvolensky, & Bernstein, 2010) that people who suffer from a low tolerance for pain not only have painful experiences but also expand the conditions that cause pain and respond to other people and the environment. If these reactions are abnormal behaviors, they can cause harm to themselves, their family, and society.

Mora, Segovia, Del, de Blas, and Garrido (2012), Vaessen, Hernaus, Myin‐Germeys, and van Amelsvoort (2015), and Bosker, Neuner, and Shah (2017) conducted experiments on the release of dopamine under pressure or stress responses. The results showed that neurotransmitters can affect people's stress response, and different pressures can lead to changes in dopamine levels. However, there are individual differences.

Cabib, Campus, and Colelli (2012), Zannas, Wiechmann, Gassen, and Binder (2016), McEwen et al. (2015) and others have performed experiments on human genetics and stress response. The results showed that genetics influences the human stress response. Genetic polymorphism and epigenetics participate in the stress response, resulting in significantly different human responses to stress.

In summary, given that there is no defined concept of psychological endurance levels or measurement methods and tools, this study first proposes the concept of the psychological endurance threshold (PET) for the first time and defines it as “the psychological state threshold of human objective physiological characteristics and outbreaks of abnormal behavior led by subjective cognitive level difference”. We conduct experiments including the construction of a psychological endurance threshold measurement table (PETMT), experience evaluation, and dopamine and gene detection to explore the existence and different levels of PET and measurement methods and tools for PET. Understanding PET will enable people to make self‐adjustment or to intervene by professionals as soon as possible and in a timely manner in the face of various negative stimuli in work and life, especially for people with lower PET, people should intervene as early as possible to reduce the harm to the individual, family, and society.

## 2 METHODS

### 2.1 Construction of PETMT

#### 2.1.1 Test subjects

The test subjects were people with normal behavior and abnormal behavior. Two groups of 9,120 volunteers between the ages of 18 and 60 (50% males and 50% females) participated in the experiment. Consents were obtained for experimentation with human subjects. The study materials and procedures were approved by Review Board of Chinese Academy of Sciences and Beijing Normal University. The experiment has been carried out in accordance with The Code of Ethics of the World Medical Association (Declaration of Helsinki) for experiments involving humans.

The normal behavior group was the population currently living under normal social conditions in China (provinces, municipalities, and autonomous regions), including farmers, workers, soldiers, doctors, nurses, teachers, students, scientific and technical personnel, athletes, civil servants, and company employees, as well as some foreign people who worked, studied, and lived in China (e.g., working in Chinese institutions, international students). This population may include some individuals who have encountered negative stimuli or have experienced abnormal behaviors and have caused harm due to reaching or exceeding PET but who had not yet been convicted by the court and served their sentences as well as people who had served a prison sentence and had been released from prison. Since the current technical means cannot accurately distinguish such people, but because it is a large population, such people don't affect the overall result, so the study still assigned these individuals to the normal behavior group.

The abnormal behavior group included Chinese people (provinces, municipalities and autonomous regions) and foreign nationality people (other countries and regions) who had been convicted of criminal offenses by the court and were serving sentences in nine prisons in Guangdong Province, China. Criminal offenders who are currently serving prison sentences have experienced some negative stimuli or have demonstrated abnormal behaviors due to various reasons that have reached or exceeded PET. They have not only caused harm to themselves, others, families, and society but also violated the law, were convicted by a court and served a prison sentence. Thus, people who demonstrate this abnormal behavior are suitable as the control group.

#### 2.1.2 Building topics and data acquisition

Psychology research mainly uses psychological measurement, experience evaluation, biochemical indicator detection, brain wave detection, eye movement analysis, nuclear magnetic resonance detection etc. For the accuracy of the study, we used a large population to build a model, so we chose a simple and accurate PETMT. The research group collected negative stimuli content to build topics. Topics were mainly collected through interviews and seminars with people in North China, Northeast China, East China, South China, Northwest China and Southwest China. Topics involved fair treatment, prices and taxes, food security, unemployment protection, compensation for house demolition, land acquisition compensation, environmental pollution, emotional communication, relationships between men and women, education costs, medical security, and public security status. After an initial screening, 1,026 topics were initially established. After small‐ and medium‐scale sample testing and dimension structure exploration (Table 1), a table with a total of 15 topics was finally formed (Table 2).

**Table 1:** Table of all tests

	Normal behavior people				Abnormal behavior people				Topic (number)
	Male		Female		Male		Female		
	Issue	Valid	Issue	Valid	Issue	Valid	Issue	Valid	
1	130	109	130	117	130	127	130	129	1,026
2	150	141	150	136	150	138	150	143	862
3	150	147	150	145	150	145	150	148	486
4	150	147	150	146	150	148	150	149	286
5	400	385	400	391	400	383	400	396	60
6	1,300	1,237	1,300	1,247	1,300	1,186	1,300	1,273	15
Total	2,280	2,164	2,280	2,183	2,280	2,127	2,280	2,238	

**Table 2:** Psychological endurance threshold measurement table

Topic	Recode	Content	Dimensions 1	Dimensions 2	Dimensions 3	Commonality
i3	A3	How long after waiting for someone will I no longer wait?	0.741			0.553
i5	A5	How many people surround to watch with unknown reason will I go to see?	0.719			0.558
i6	A4	If no one collects the garbage, how long will it take for me to throw garbage everywhere?	0.671			0.487
i1	A1	How many people would have to resign because of a bad boss before I will quit?	0.642			0.434
i2	A2	How long would I have to check lots of other people's money every day before I would want to take the money as my own?	0.639			0.436
i9	B4	How much lower would the purchase price of grain have to be compared to last year for me to feel uncomfortable?		0.751		0.588
i7	B2	How much would the price index of living have to rise in a year for me to complain?		0.718		0.566
i10	B5	How much smaller would the purchase area have to be than stated in a contract for me to not accept it?		0.702		0.526
i8	B3	How much of education costs accounting for annual income would I find unbearable?		0.682		0.604
i4	B1	How much would the price of a valuable item just bought have to fall to make me feel a great loss?		0.583		0.414
i15	C5	How many times after the government's land acquisition and demolition compensation consultation could not reach an agreement would I be dissatisfied?			0.659	0.492
i16	C3	How many security incidents happening each month at my residence would cause me anxiety?			0.64	0.446
i14	C1	After how many times of being forced to go on blind dates in a month would I refuse?			0.623	0.402
i11	C2	How many unfair things that I am forced to do would be unbearable?			0.567	0.344
i12	C4	How many days after setting up a roadblock complaint would I be annoyed if no one handles it?			0.518	0.351

#### 2.1.3 Statistics

The test data were input in EXCEL and analyzed with SPSS13.0. We used critical ratios (CR values), eigenvalues >1 dimension extraction, principal component method factor analysis, reliability testing, validity testing, differential ANOVA, MANOVA (*F* value) and regression analysis.

### 2.2 Application of PETMT

#### 2.2.1 Test subjects

After thoroughly observing various social strata for many years, we believe that under various stress situations such as fight and suicide, it is impossible for people under stress to cooperate with the research team to complete PETMT measurement, dopamine level detection etc., while pregnant women suffer from abnormal pain during the delivery process and can cooperate with researchers; the research group believes that the speech sound content and physical movements of the pregnant women during the delivery process involve an instinctual physical stress response. Thus, pregnant women during the delivery process are currently the most suitable research population for the examination of PET during stress response. To reduce the interference of various factors, especially drug interference, pregnant women in this study were required to be 20–30 years old, pregnant with their first child, at 34–44 weeks of pregnancy, vaginal delivery finally, with no comprehensive symptoms and no history of drug use during childbirth. A total of 66 pregnant women who fulfilled the above conditions registered on the basis of voluntary enrollment were recruited for the experiment. The study materials and procedures were approved by Review Board of Southern Medical University. All procedures performed in studies involving human participants were in accordance with the 1964 Helsinki Declaration and its later amendments.

#### 2.2.2 Data acquisition

Given that pregnant women during the delivery process are already under stress, our research cannot add any additional stimuli and cannot interfere with their delivery process or cause any trauma, so we choose the simple, accurate, and acceptable PETMT. During the delivery process, the pregnant women completed the PETMT measurement orally. A nurse read the questions, the pregnant women answered the options orally, and the nurse filled out the form.

#### 2.2.3 Statistics

Same as Method 2.1.3.

### 2.3 Experience evaluation

#### 2.3.1 Test subjects

Same as Method 2.2.1.

#### 2.3.2 Evaluation criteria

Given that pregnant women during the delivery process are already under stress, our research cannot add any additional stimuli and cannot interfere with their delivery process or cause any trauma, so we choose the simple, accurate, and acceptable experience evaluation. Mr. QC. Jing, a member of the research team, has participated in the rapid selection of flying students in China. Using the experience of three old flying instructors to quickly select students, the project achieved very good practical results, so the experience evaluation system began to be used. However, because it is a confidential project, it has not been published yet. Based on the method used by Mr. QC. Jing, who used an experience evaluation system to quickly select flying students, a group of three senior doctors or midwives who had worked in the delivery room for 20–30 years was assigned. The doctors and midwives independently gave scores of high, medium, and low according to experience regarding the degree of physical and speech sound response of the pregnant women during the delivery process. The principle for the total score summary was as follows: if three people gave the same score, the total score was the score; if the score of two people was high and one was medium or low, the total score was still high; if one score was high and two people provided scores of medium, the total score was medium; if two people gave medium scores and one person gave a low score, the total score was still medium; if one person provided medium scores and two people provided low scores, the total score remained low.

#### 2.3.3 Statistics

The scoring system for the experience evaluation was input using EXCEL.

### 2.4 Dopamine level detection

#### 2.4.1 Test subjects

Same as Method 2.2.1.

#### 2.4.2 Data acquisition

Given that pregnant women during the delivery process are already under stress, our research cannot add any additional stimuli and cannot interfere with their delivery process or cause any trauma, so we choose the simple, accurate, and acceptable dopamine level detection. One milliliter of peripheral blood (EDTA anticoagulant) was collected from each subject during delivery. Dopamine levels were detected using a human dopamine ELISA Kit (TSZ, USA) according to the manufacturer's standard operating procedures. Blank wells were set separately, 50 µl of standards or samples was added to the appropriate well of the antibody precoated microtiter plate, and gently mixed. The plate was incubated for 45 min at 37°C. Liquid was removed, dried by swing, washing buffer was added to every well, stilled for 30 s then removed, repeated four times. Fifty microliter diluted biotinylated anti‐IgG was added to all wells, and incubated for 30 min at 37°C. Washing was carried out using the abovementioned procedure, 50 µl streptavidin‐HRP was added to all wells, gently mixed and incubated for 15 min at 37°C. Washing was carried out using the abovementioned procedure. Fifty microliter chromogen solution A and chromogen solution B was added to each well, incubated for 15 min at 37°C. Fifty microliter stop solution was added to each well to stop the reaction (the blue color changed to yellow color immediately). Blank well was taken as zero, the optical density (OD) at 450 nm was measured after adding stop solution within 15 min. The duplicate readings for each standard, control, and sample were averaged. A standard curve was constructed by plotting the mean absorbance for each standard on the y‐axis against the concentration on the x‐axis and a best fit curve was drawn through the points on the graph. If samples have been diluted, the concentration read from the standard curve must be multiplied by the dilution factor. The experiment was repeated three times with three replicates per sample. In the plates detected in the same batch, one plate was randomly taken, and six different samples were selected to calculate intra‐assay %CV. In each of the three different batches of plates, one plate was randomly taken, and six different samples were selected to calculate inter‐assay %CV. Intra‐assay %CV was less than 10%, inter‐assay %CV was less than 15%.

#### 2.4.3 Statistics

The data were processed using SPSS 13.0 and one‐way ANOVA.

### 2.5 Gene detection

#### 2.5.1 Test subjects

After the subjects of Method 2.2.1 were tested for dopamine detection, experience evaluation and PETMT measurement, five individuals were randomly selected from the high PET group and five from the low PET group, for a total of 10 subjects.

#### 2.5.2 Genetic testing

Given that pregnant women during the delivery process are already under stress, our research cannot add any additional stimuli and cannot interfere with their delivery process or cause any trauma, so we choose the simple, accurate, and acceptable genetic testing. In order to reflect the physiological characteristics of PET more accurately, we used the method of genetic testing while detecting the dopamine level to clarify the relationship between PET and human genes at the molecular level. First, 1 ml peripheral blood (EDTA anticoagulant) was collected from each pregnant woman during the delivery process and transferred to PAXgene blood collection tubes (QIAGEN, Germany). Total RNA was extracted and purified using PAXgene Blood RNA Kit (Cat#762174, QIAGEN, GmBH, Germany) following the manufacturer's instructions and checked for a RIN number to inspect RNA integration by an Agilent Bioanalyzer 2,100 (Agilent technologies, Santa Clara, CA). Total RNA was amplified, labeled and purified by using GeneChip3' IVT PLUS Reagent Kit (Cat#902416, Affymetrix, Santa Clara, CA) followed the manufacturer's instructions to obtain biotin‐labeled cDNA. Array hybridization and washing were performed using GeneChip® Hybridization, Wash and Stain Kit (Cat#900720, Affymetrix) in Hybridization Oven 645 (Cat#00‐0331‐220V, Affymetrix) and Fluidics Station 450 (Cat#00‐0079, Affymetrix) following the manufacturer's instructions. Slides were scanned by GeneChip® Scanner 3,000 (Cat#00‐00212, Affymetrix) and Command Console Software 4.0 (Affymetrix) with default settings. Raw data were normalized by MAS 5.0 algorithm, Affy packages in R.

#### 2.5.3 Quantitative real‐time PCR

About 0.5 microgram of total RNA was reversed transcribed with ReverTra Ace qPCR Kit (TOYOBO, FSQ‐101). cDNA equivalent to 10 ng was used for quantitative Real‐Time PCR (qPCR) amplification (Applied Biosystems, Foster City, CA) with SYBR green PCR master mix (Applied Biosystems). The relative levels of expression of genes were normalized according to those of human β‐actin (5′‐CTGGAACGGTGAAGGTGACA‐3′, 5′‐CGGCCACATTGTGAACTTTG‐3′). qPCR data were calculated using the comparative Ct method (Applied Biosystems). Primers to the coding region of human *AKT1* (5′‐AGATGCAACCTCACTATGGTATGC‐3′, 5′‐TCCTCAGAGACACGGCCTTAG‐3′), *PRDM4* (5′‐GCAGACCCACCCTTAAGTTGAC‐3′, 5′‐GCAAAACCTTCCCACCAGAGT‐3′), *BAX* (5′‐TGTCGCCCTTTTCTACTTTGC‐3′, 5′‐CTGATCAGTTCCGGCACCTT‐3′) were used for qPCR analysis. The experimental conditions were 50°C, 2 min; 95°C, 10 min; (95°C, 15 s; 60°C, 1 min) 40 cycles. All qPCR reactions were performed in triplicate, and three independent RNA samples were assayed for each time point.

#### 2.5.4 Data processing

Fold change values for genes were calculated as the ratio of the signal values of the group with low PET compared with the group with high PET. Differentially expressed genes were screened according to a gene expression changed significantly *p* < 0.01. Then, KEGG function enrichment analysis was performed using DAVID and KEGG database. The selection criteria were the number of differentially expressed genes on a certain term >2, and *p* < 0.05.

## 3 RESULTS

### 3.1 PET can be accurately measured by PETMT

This study adopted negative stimuli of life as the content, measured PET of normal and abnormal behavior groups, explored differences in cognitive ability and effective discrimination between the two groups of people, and constructed a convenient, accurate, and effective PETMT.

**Figure 1:**
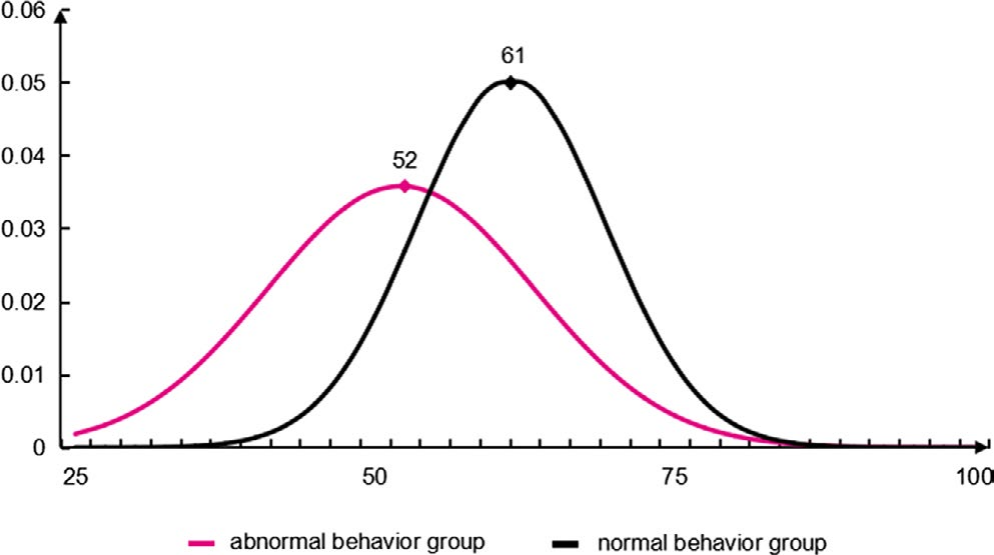
Psychological endurance threshold (PET) score curve of normal and abnormal behavior groups. PET score curve of the normal behavior group was obviously biased toward the right, and the peak was 61 points (out of 100 points). PET score curve of the abnormal behavior group was obviously biased toward the left, and the peak was 52 points (out of 100 points)

The results showed that PETMT with a total of 15 topics was finally formed with three dimensions (social, economic and living) and five topics for each dimension (Table 1). The reliability and validity of PETMT were good, and there was a significant difference between the PET of normal and abnormal behavior groups (*p* < 0.05). PET score curve of the normal behavior group was obviously biased toward the right, and the peak was 61 points (out of 100 points). PET score curve of the abnormal behavior group was obviously biased toward the left, and the peak was 52 points (out of 100 points) (Figure 1). The results indicate that people with a PET score higher than 61 points have a higher PET and tend to be individuals who are not susceptible to outbreaks of abnormal behavior. Those who score below 52 points have a lower PET and tend to be individuals who are susceptible to outbreaks of abnormal behavior.

### 3.2 Experience evaluation is highly consistent with the measurement results of PETMT

After thoroughly observing various social strata for many years, the research group believes that the speech sound content and physical movements of pregnant women during the delivery process involve an instinctual physical stress response. Thus, pregnant women during the delivery process are currently the most suitable research population for the examination of PET during stress response. We conducted PETMT and an experience evaluation on the process of maternal childbirth to explore the PET of pregnant women under stress conditions and the relationship between the two experiments.

**Figure 2:**
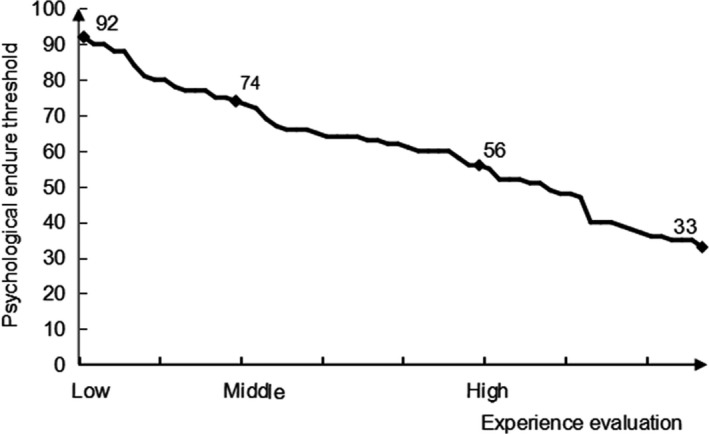
Results of experience evaluation and psychological endurance threshold measurement table (PETMT) during the delivery process of pregnant women. Experience evaluation during the delivery process of pregnant women was negatively correlated with PETMT results. Pregnant women during the delivery process who have lower scores of experience evaluation have a higher psychological endurance threshold (PET) detected by PETMT, whereas those with higher scores of experience evaluation have a lower PET detected by PETMT

The results showed that the experience evaluation during the delivery process of pregnant women was negatively correlated with the results of PETMT (Figure 2). The results indicate that pregnant women during the delivery process who have lower scores of experience evaluation have a higher PET detected by PETMT, whereas those with higher scores of experience evaluation have a lower PET detected by PETMT.

### 3.3 Dopamine level is highly correlated with the results of experience evaluation and PETMT

Based on the dopamine detection and experience evaluation, and PETMT in the process of maternal delivery, we explored the dopamine level released in pregnant women under stress response as well as the relationship between dopamine level and experience evaluation, and PETMT.

**Figure 3:**
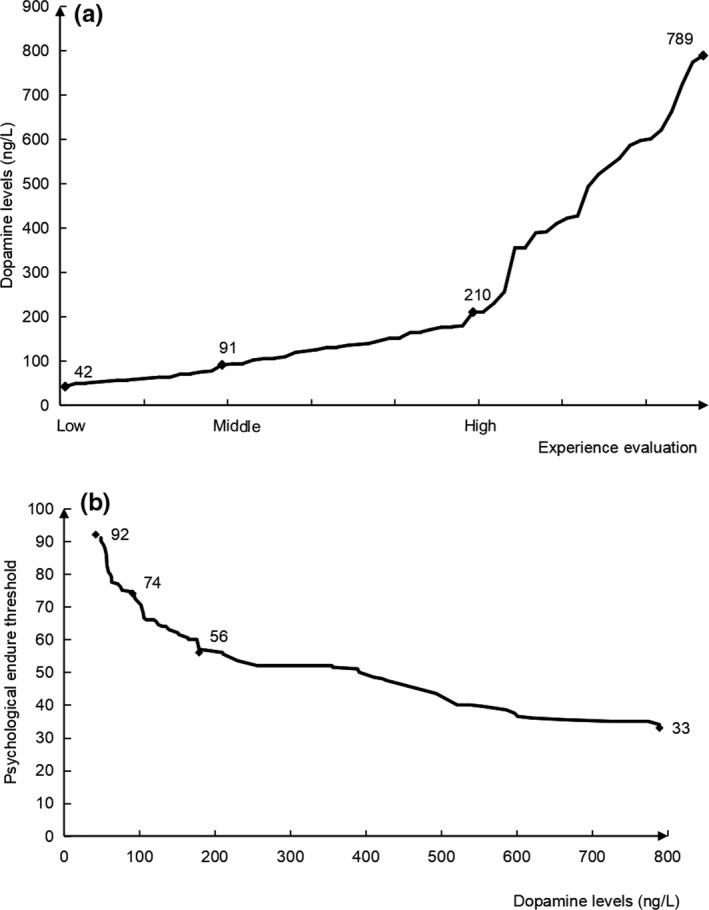
Results of dopamine, experience evaluation and psychological endurance threshold measurement table (PETMT) in the delivery process of pregnant women. The dopamine results were positively correlated with the experience evaluation results (a) and negatively correlated with the PETMT results (b). Pregnant women in the process of maternal delivery who have lower dopamine levels have lower experience evaluation results and a higher psychological endurance threshold (PET) detected by PETMT. In contrast, those with higher dopamine levels have higher experience evaluation results and a lower PET detected by PETMT

The results showed that the release levels of dopamine significantly changed during the delivery process of pregnant women. The dopamine results were positively correlated with the experience evaluation results (Figure 3a), and negatively correlated with the PETMT results (Figure 3b). The results indicate that pregnant women in the process of maternal delivery who have lower dopamine levels have lower experience evaluation results and a higher PET detected by the PETMT. In contrast, those with higher dopamine levels have higher experience evaluation results and a lower PET detected by the PETMT.

### 3.4 Gene detection first finds genetic markers related to PET

Genes are genetic material carried by human beings. The research group assumes that a low PET or a tendency toward aggressive outbreaks may be related to people's specific genes. We used whole genome detection and functional analysis during the maternal delivery of pregnant women combined with dopamine detection, experience evaluation, and PETMT to explore whether this hypothesis was established.

**Figure 4:**
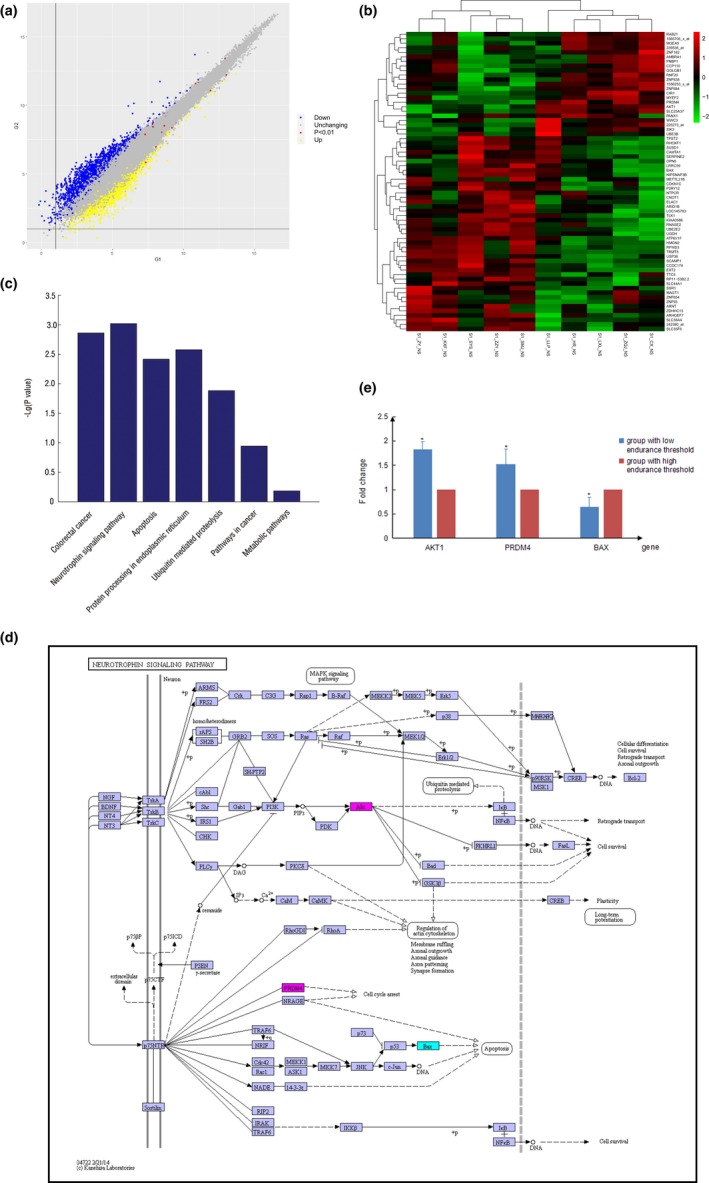
Results of whole genome detection and analysis in pregnant women during maternal delivery. The gene expression levels in the group with low PET compared with the group with high PET were significantly different, and there were 66 differentially expressed genes of 38,500 genes (a), 23 genes were upregulated and 43 genes were downregulated (b). Differentially expressed genes were mainly involved in seven signaling pathways, of which the neurotrophin signaling pathway changed most significantly (c). Expressions of *AKT1*, *PRDM4* and *BAX* in neurotrophin signaling pathway were significantly changed. Magenta represents upregulation of gene expression and cyan represents downregulation of gene expression. AKT1 and PRDM4 were significantly downregulated and BAX was significantly upregulated in people with a high PET; AKT1 and PRDM4 were significantly upregulated and BAX was significantly downregulated in people with a low PET (d). The results of qPCR showed the expression of three genes were significantly different in groups with a high and low PET(*<0.01), AKT1 and PRDM4 were significantly upregulated and BAX was significantly downregulated in low PET group compared with high PET group (e). [Correction added on 24 May 2019, after first online publication: in Figure 4d, the gene “SC‐1” has been changed to “PRDM4”.]

The results of whole genome detection, dopamine level, experience evaluation, and PETMT during maternal delivery of pregnant women were highly consistent. The gene expression levels in the group with low PET compared with the group with high PET were significantly different, and there were 66 differentially expressed genes of 38,500 genes (Figure 4a), 23 genes were upregulated and 43 genes were downregulated (Figure 4b). KEGG function enrichment analysis of differentially expressed genes using DAVID and KEGG database revealed that the differentially expressed genes were mainly involved in seven signaling pathways, of which the neurotrophin signaling pathway changed most significantly (Figure 4c). In the neurotrophin signaling pathway, expressions of *AKT1*, *PRDM4* and *BAX* were significantly changed (Figure 4d). *AKT1* and *PRDM4* were significantly downregulated and *BAX* was significantly upregulated in people with a high PET; *AKT1* and *PRDM4* were significantly upregulated and *BAX* was significantly downregulated in people with a low PET.

The expression of *AKT1*, *PRDM4* and *BAX* was verified by qPCR. It was confirmed that the expression of three genes were significantly different in groups with a high and low PET; *AKT1* and *PRDM4* were significantly upregulated and *BAX* was significantly downregulated in low PET group compared with high PET group (Figure 4e).

## 4 DISCUSSION

In this study, we found that PET exists objectively and can be measured exactly through experiments including the PETMT, experience evaluation, dopamine and gene detection which complement each other. The first PETMT can quantify the PET. We also found that PET is determined by three genes *AKT1*, *PRDM4,* and *BAX*.

Neurotrophic factor is a class of proteins that have a specific role in the nervous system and promote neuronal differentiation, survival, neurite outgrowth, synapse formation, and cell migration and proliferation (Hempstead, 2015, p. 9–19). Neurotrophic factors activate many complex signaling pathways by activating two different types of receptors, the Trk family receptor and the p75 receptor, and ultimately exert biological effects (Skaper, 2012, p. 1–12). The Trk receptor and the p75 receptor interact to maintain a balance between neuronal survival and death.

The Trks receptor activates the PI3K/AKT pathway, which phosphorylates a variety of molecules, especially the proapoptotic protein Bad, thereby inhibiting its apoptosis‐inducing properties and promoting neuronal survival. *AKT1*, serine/threonine kinase 1，is a critical mediator of growth factor‐induced neuronal survival in the developing nervous system. Survival factors can suppress apoptosis by activating *AKT1*. This study found that *AKT1* expression was significantly upregulated in the low PET group compared with the high PET group, indicating that it can promote the phosphorylation of the apoptotic protein Bad, thereby inhibiting its apoptosis‐inducing properties and promoting neuronal survival.

Activation of p75 receptor can regulate neuronal survival and death through two pathways. One is JNK‐P53‐*BAX* pathway, and the signaling molecule *BAX,* which is BCL2‐associated X, apoptosis regulator, is one of the most representative proapoptotic genes, activation of this pathway promotes neuronal apoptosis. This study found that *BAX* expression was significantly downregulated in the low PET group compared with the high PET group, indicating that the pathway was inhibited, leading to inhibition of apoptosis and promoting neuronal survival. Another pathway affects the cell cycle arrest through the cell cycle regulatory molecule *PRDM4*, resulting in inhibition of cell growth, thereby reducing neuronal production. *PRDM4* is PR/SET domain 4 and involved in cell differentiation. This study found that *PRDM4* was significantly upregulated in the low PET group compared with the high PET group, indicating that it promoted cell cycle arrest, inhibited cell growth, and led to a decrease in new neuronal production.

In summary, this study found that *AKT1* was significantly upregulated and *BAX* was significantly downregulated in the low PET group compared with the high PET group, resulting in inhibition of neuronal apoptosis and promotion of neuronal survival; *PRDM4* was significantly upregulated, resulting in promotion of neuronal cell cycle arrest, suppression of new neuronal growth. The results indicate that in the period of neuron occurrence, the interaction between the above two can increase the survival of unsound neurons and reduce the production of new neurons; after the neurons mature, the interaction between the above two can increase the survival of aging neurons. It eventually leads to a decrease in the production of new neurons, increase in the number of unsound and aging neurons, an abnormal number of neurons and a metabolic abnormality, which ultimately lead to low PET and prone to abnormal behavior.

The significance of this study is to discover people with abnormal expression of *AKT1*, *PRDM4*, and *BAX* have lower PET and tend to commit abnormal behavior more easily. Understanding PET will enable people to make self‐adjustment or to intervene by professionals as soon as possible and in a timely manner in the face of various negative stimuli in work and life, especially for people with lower PET, people should intervene as early as possible to reduce the harm to the individual, family, and society.

## CONFLICT OF INTEREST

The author declares no conflicts of interest.

## AUTHOR CONTRIBUTIONS

ZX fully presided over and conducted the entire process. QJ participated in the whole process and focused on constructing the PETMT and the experience evaluation systems as well as the planning of dopamine and gene research. HZ participated in the whole process and focused on constructing the PETMT. YL participated in the whole process and focused on dopamine and gene detection work.

## INFORMED CONSENT

Written informed consent was obtained from adults (parents or guardians of minor children and participants 18 years of age or older) and assent was obtained from minor children whose parents or guardians provided written consent.

### DATA AVAILABILITY STATEMENT

The data that support the findings of this study are available from the corresponding author upon reasonable request.
